# Synthesis and Characterization of Magnesium Oxide-Enhanced Chitosan-Based Hemostatic Gels with Antibacterial Properties: Role of Amino Acids and Crosslinking

**DOI:** 10.3390/molecules30071496

**Published:** 2025-03-27

**Authors:** Julia Radwan-Pragłowska, Paulina Bąk, Łukasz Janus, Aleksandra Sierakowska-Byczek, Piotr Radomski, Agnieszka Kramek, Justyna Gumieniak, Dariusz Bogdał

**Affiliations:** 1 Department of Biotechnology and Physical Chemistry, Faculty of Chemical Engineering and Technology, Cracow University of Technology, Warszawska 24 Street, 31-155 Cracow, Poland; paulinabk63@gmail.com (P.B.); lukasz.janus@pk.edu.pl (Ł.J.); a.sierakowska3530@doktorant.pk.edu.pl (A.S.-B.); dariusz.bogdal@pk.edu.pl (D.B.); 2 Department of Chemical Technology and Environmental Analytics, Faculty of Chemical Engineering and Technology, Cracow University of Technology, Warszawska 24 Street, 31-155 Cracow, Poland; piotr.radomski@pk.edu.pl; 3 The Faculty of Mechanics and Technology, Rzeszow University of Technology, ul. Kwiatkowskiego 4, 37-450 Stalowa Wola, Poland; a.kramek@prz.edu.pl (A.K.); j.gumieniak@prz.edu.pl (J.G.); 4 Department of Component Manufacturing and Production Organization, Rzeszów University of Technology, 37-450 Stalowa Wola, Poland

**Keywords:** natural compounds, chitosan biomaterials, hemostatic agents, antibacterial effect

## Abstract

Excessive blood loss is a leading cause of mortality among soldiers and accident victims. The wound healing process typically ranges from three weeks to several months, with disruptions in healing stages potentially prolonging recovery time. Chronic wounds may persist for years, creating a favorable environment for microbial growth. Chitosan, a derivative of chitin—the second most abundant biopolymer in nature—is obtained through deacetylation and exhibits mucoadhesive, analgesic, antioxidant, biodegradable, non-toxic, and biocompatible properties. Due to its hemostatic and regenerative support capabilities, chitosan is widely applied in the food, cosmetic, and agricultural industries; environmental protection; and as a key component in dressings for chronic wound healing. Notably, its antibacterial properties make it a promising candidate for novel biomaterials to replace traditional antibiotics and prevent the emergence of drug-resistant strains. The primary aim of this study was the chemical cross-linking of chitosan with the amino acids L-aspartic and L-glutamic acid in the presence of periclase (magnesium oxide) under microwave radiation conditions. Subsequent research stages involved the analysis of the samples’ physicochemical properties using SEM, FT-IR, XPS, atomic absorption spectrometry, swelling behavior (in water, SBF, and blood), porosity, and density. Biological assessments included biodegradation, cytotoxicity, and antibacterial activity against Escherichia coli and Staphylococcus aureus. The obtained results confirmed the high potential of the newly developed hemostatic agents for effective hemorrhage management under non-sterile conditions.

## 1. Introduction

The skin serves as a critical defensive barrier, providing thermal insulation, retaining fluids, and protecting against pathogens that could otherwise enter the body from the external environment [1,2]. The process of wound healing following an injury is highly complex, involving a precisely coordinated cascade of biological events that must occur in a specific sequence and timeframe. Repair begins immediately after a wound is inflicted, with hemostasis being one of the initial stages. This step is crucial for stopping bleeding, which commonly results from traumatic events such as vehicular accidents, surgical procedures, or combat injuries. Uncontrolled hemorrhage in such cases poses a significant challenge for first responders and medical professionals, particularly in pre-hospital care settings, where rapid intervention is essential to prevent excessive blood loss. Hemorrhage remains one of the leading causes of mortality at the scene of an incident—accounting for approximately 50% of fatalities in accidents and over 90% in trauma cases involving soldiers [1,2,3,4].

Once hemostasis is achieved, subsequent stages of tissue repair can proceed. However, external factors such as the presence of foreign bodies, bacterial infections, and pre-existing chronic diseases can severely impact the wound healing process, leading to delayed recovery or even the development of chronic wounds. These wounds, categorized as non-healing or hard-to-heal, are often associated with conditions such as cardiovascular diseases, diabetes, and cancer, which are becoming increasingly prevalent worldwide. It is estimated that by 2026, chronic diseases will affect between 20 and 60 million people globally, further escalating the incidence of chronic wounds. Surgical interventions are frequently required to treat such conditions; however, post-surgical recovery often involves prolonged hospitalization, increasing the risk of complications, including wound infections and chronic wound formation. Additional risk factors such as patient age, lifestyle, and comorbidities further contribute to impaired wound healing [1,2,5].

Current treatment strategies for wound management, particularly chronic wounds, are often inadequate and highly dependent on daily wound care and close monitoring. Standard approaches involve wound debridement to remove necrotic or infected tissue, frequent dressing changes to manage exudate, and maintaining a moist environment to promote healing. Despite these efforts, the risk of severe complications—including infections, sepsis, limb amputations, and even mortality—remains high [1,2]. Consequently, there is a pressing need to develop more effective, multifunctional wound dressings with enhanced hemostatic, antimicrobial, and regenerative properties.

Chitosan, a natural polysaccharide derived from the deacetylation of chitin, has emerged as a promising biopolymer for wound dressing applications due to its hemostatic properties and ability to support tissue regeneration [6,7,8,9,10]. Its biological activity is largely attributed to its polycationic nature, which enables interactions with negatively charged bacterial cell walls, leading to bacterial membrane disruption and lysis. Additionally, chitosan’s amino groups can bind to negatively charged bacterial DNA, inhibiting genetic material synthesis and ultimately resulting in bacterial cell death. Chitosan also enhances macrophage activity, indirectly reinforcing its antibacterial properties by aiding in the clearance of pathogens from the body. These characteristics make chitosan a highly attractive candidate for medical applications aimed at preventing bacterial infections and promoting wound healing [10,11,12,13,14,15].

However, while chitosan exhibits numerous beneficial properties, its use alone may be insufficient for advanced wound care applications. It has been reported that unmodified chitosan can cause chronic inflammation and lacks the necessary mechanical strength for long-term applications. Therefore, various chemical modifications are often applied to enhance its properties. These modifications can influence chitosan’s biological activity, as well as its interactions with cells and surrounding tissues. To further improve its performance, modern chitosan-based materials are increasingly being functionalized with carefully selected additives that enhance their mechanical strength, antibacterial properties, and bioactivity. Among these additives, ceramic nanomaterials have attracted considerable attention.

Periclase (magnesium oxide, MgO) is one such ceramic material with a range of desirable properties, including chemical inertness, corrosion resistance, and excellent thermal stability. It is also hygroscopic and exhibits outstanding electrical and optical characteristics. As a refractory material, periclase aligns with green chemistry principles due to its environmental safety and minimal ecological impact. These properties have led to widespread use in industries such as automotive, aerospace, and metallurgy, as well as in the production of paints, substrates, superconductors, and refractory materials. In the chemical industry, periclase functions as a catalyst for organic reactions, a biosensor, an adsorbent, and a sensor for detecting heavy metals in water and wastewater treatment, particularly in textile industry effluents [16,17,18,19,20,21,22,23,24].

Beyond its industrial applications, periclase has demonstrated significant potential in biomedical applications due to its biocidal capabilities. Studies suggest that MgO can generate reactive oxygen species (ROS), such as superoxide anions (•O_2_^−^), which can cause oxidative damage to bacterial DNA and proteins, contributing to its antimicrobial activity. This property makes periclase a valuable additive for biomaterials with antibacterial functions, including potential applications in anticancer therapies. Additionally, chitosan-based biomaterials containing MgO have shown enhanced antibacterial efficacy compared to chitosan alone, while also mitigating cytotoxic effects on healthy cells. Moreover, MgO-based ceramic materials exhibit excellent biocompatibility and mechanical strength, making them particularly suitable for applications such as bone implants. Upon degradation, periclase releases magnesium ions, which play a vital role in numerous physiological processes and act as essential cofactors for enzymatic reactions. As the fourth most abundant element in the human body, magnesium is non-toxic and essential for cellular function, further supporting the suitability of MgO-based materials for biomedical use [20,21,22,23,24,25,26].

The development of advanced chitosan-based biomaterials requires not only the careful selection of functional additives but also the optimization of processing methods to ensure high purity, stability, and efficacy. Chitosan processing can be challenging due to its limited solubility, low reactivity, and poor thermal stability. Additionally, conventional chemical modification techniques may involve the use of harsh solvents or catalysts that could compromise the biocompatibility of the final product. To overcome these limitations, microwave-assisted synthesis has emerged as a promising strategy for modifying chitosan-based biomaterials. Microwave radiation offers several advantages, including significantly reduced reaction times, elimination of toxic catalysts, lower Gibbs energy requirements, and improved reaction efficiency, making it an environmentally friendly and cost-effective approach [27,28,29,30,31,32,33,34].

The primary objective of this study is to synthesize chemically cross-linked chitosan matrices incorporating selected antibacterial agents under microwave-assisted conditions and to evaluate their physicochemical and biological properties. The scope of the research includes the preparation of cross-linked chitosan matrices using microwave irradiation, the incorporation of antibacterial agents, and comprehensive physicochemical characterization using Fourier-transform infrared spectroscopy (FT-IR), X-ray photoelectron spectroscopy (XPS), and scanning electron microscopy (SEM). Further assessments include swelling behavior analysis, biodegradation studies, antibacterial activity testing against selected Gram-positive and Gram-negative bacterial strains, and cytotoxicity evaluation using the L929 fibroblast cell line. The findings of this study will provide valuable insights into the potential of these novel chitosan-based biomaterials for hemostatic applications, particularly in hemorrhage management under non-sterile conditions.

## 2. Results and Discussion

**Figure 1:**
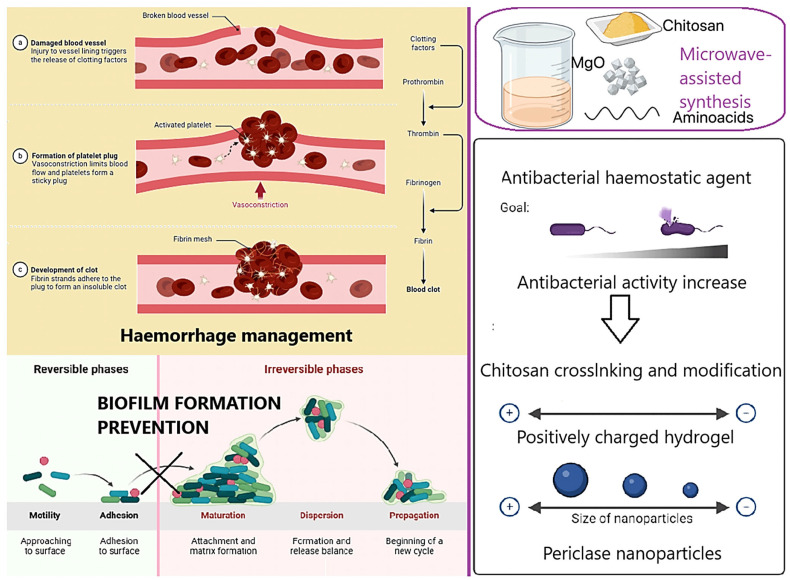
General scheme of potential hemostatic agents’ preparation and antibacterial properties enhancement.

Figure 1 presents the general aim of the following research. The main scientific hypothesis was to increase the antibacterial properties of the native fungal chitosan as a result of chemical crosslinking using two amino acids without losing free amino groups and synergistic effects with periclase nanoparticles—highly crystalline magnesium oxide. Fungal chitosan presents several advantages over marine-derived chitosan, making it a superior candidate for hemostatic applications. Its production through controlled fermentation ensures higher purity, eliminating endotoxins and allergens commonly found in shellfish-derived chitosan, thus improving biocompatibility and reducing the risk of immune reactions. Additionally, fungal chitosan exhibits enhanced solubility at physiological pH, facilitating better bioavailability and interaction with biological systems. Its more uniform molecular weight and degree of deacetylation contribute to improved antimicrobial properties and cell adhesion, which are critical for wound healing and infection control. Moreover, fungal chitosan is sustainably sourced from renewable biomass, offering an ethical and consistent alternative to marine-based production. These properties collectively enhance its effectiveness in hemostatic materials by promoting rapid clot formation, reducing inflammation, and supporting tissue regeneration. The addition of periclase (MgO) enhances the properties of chitosan-based hemostatic agents by addressing key limitations associated with pure chitosan. One of the primary benefits is pH modulation, as periclase acts as an alkaline buffer, counteracting the localized acidity that can arise from chitosan degradation. This is particularly important for maintaining a physiological pH in wound environments, reducing the risk of tissue irritation and inflammatory responses. Additionally, periclase contributes to antimicrobial activity, as MgO nanoparticles generate reactive oxygen species (ROS) that disrupt bacterial membranes, enhancing the infection-preventive properties of the hemostatic material.

From a structural perspective, the incorporation of periclase improves the mechanical strength of chitosan-based hydrogels and scaffolds, making them more durable while maintaining flexibility for effective wound coverage. Furthermore, periclase releases bioactive magnesium ions, which play a crucial role in cell proliferation, tissue regeneration, and coagulation processes. Magnesium ions activate platelets and improve fibrin network formation, accelerating clot formation and promoting faster healing. The combined effects of pH regulation, antimicrobial activity, mechanical reinforcement, and bioactivity make periclase an excellent additive for enhancing the performance of chitosan-based hemostatic materials.

### 2.1. Periclase Characteristics

**Figure 2:**
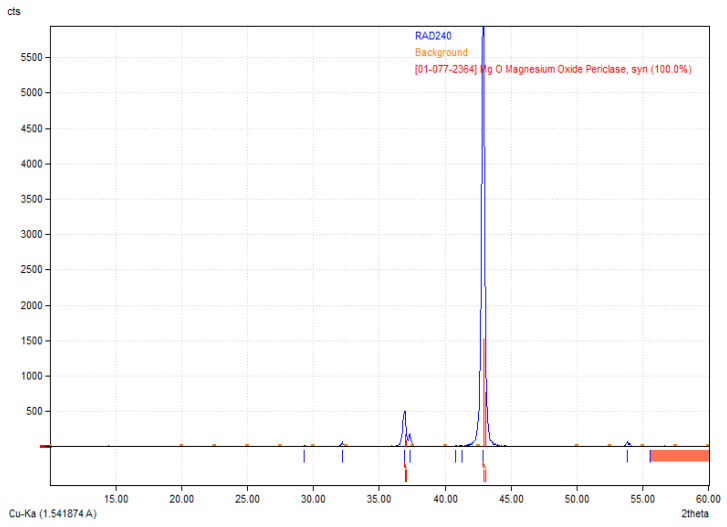
XRD diffractogram of periclase.

Figure 2 reveals the crystalline structure of the obtained MgO nanoparticles. The X-ray diffraction (XRD) pattern presented corresponds to periclase (MgO), as confirmed by the reference diffraction pattern [01-077-2364] [35,36]. The diffractogram exhibits characteristic sharp peaks, indicating a well-crystallized structure. The most intense peak appears around 42.9° 2θ, corresponding to the (200) crystallographic plane of MgO, which is a dominant feature of periclase. Additional peaks observed in the range of 35° to 50° further confirm the presence of cubic MgO. The high intensity of the primary diffraction peak suggests that the sample is highly crystalline, with minimal amorphous content. The absence of additional unidentified peaks indicates phase purity, meaning no significant secondary phases or impurities were detected. The relatively low background signal also supports the conclusion that the material is well-ordered and has minimal structural defects. These results validate the successful synthesis or presence of MgO periclase in the sample, which is relevant for applications where high crystallinity and phase purity are essential, such as in biomaterials.

**Figure 3:**
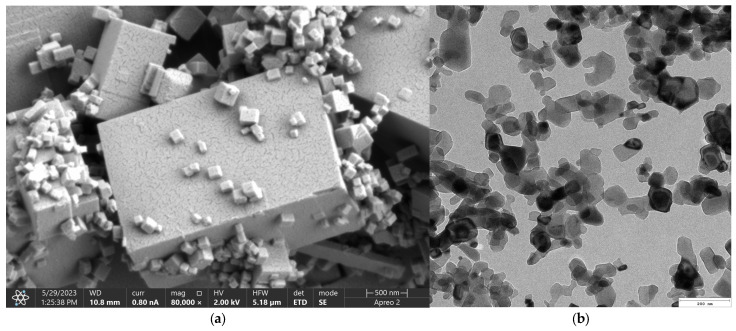
(**a**)—SEM; (**b**)—TEM microphotograph of periclase nanoparticles.

Figure 3 presents microphotographs of the MgO ((a)—SEM and (b)—TEM, respectively). The SEM micrograph of MgO powder (Figure 3a) reveals a well-defined crystalline morphology, indicative of its high degree of crystallinity. The particles exhibit a cubic structure, consistent with the characteristic morphology of periclase. The surface of the particles appears smooth with sharp edges, confirming the presence of highly ordered crystal planes. Particle sizes are relatively uniform, which agrees with previous reports on MgO periclase synthesis. Additionally, agglomeration is minimal, suggesting a high purity of the synthesized material and an absence of significant structural defects. The high crystallinity observed in the SEM images correlates with the material’s inherent thermal stability and mechanical properties, making it suitable for applications requiring structural integrity and resistance to degradation. The well-defined edges and uniform morphology also indicate a controlled synthesis process, ensuring reproducibility for further applications. The TEM images provide further insight into the microstructural features of MgO periclase. The lattice fringes observed in high-resolution TEM (Figure 3b) images confirm the highly crystalline nature of the material. Moreover, the TEM analysis highlights the absence of significant defects, dislocations, or amorphous regions, reinforcing the findings from SEM. The uniformity in particle distribution and crystallinity suggests that the synthesis method employed effectively controls nucleation and growth, resulting in a material with excellent structural homogeneity. These structural characteristics of MgO periclase have significant implications for its applications in catalysis, biomedicine, and refractory materials. The high crystallinity ensures optimal thermal and chemical stability, while the well-defined morphology enhances its reactivity in surface-related applications. The absence of amorphous phases also indicates minimal impurities, further confirming the high-quality synthesis of the material.

### 2.2. Weight Swelling Properties

**Figure 4:**
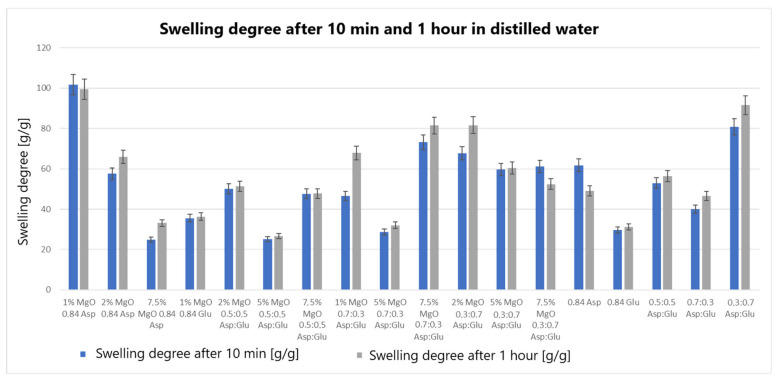
Swelling abilities of prepared samples in distilled water.

**Figure 5:**
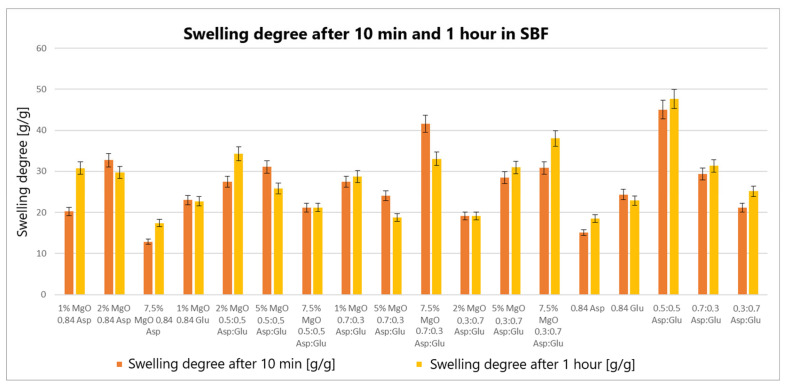
Swelling abilities of prepared samples in SBF.

**Figure 6:**
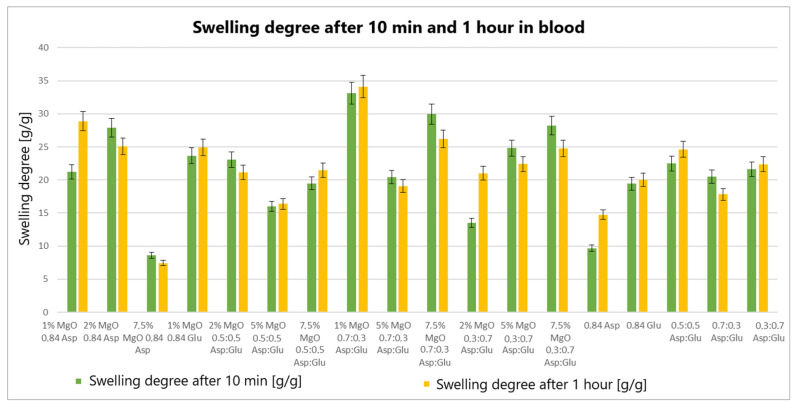
Swelling abilities of prepared samples in blood.

The swelling properties of chitosan-based hydrogels containing periclase (MgO) were compared to those of reference samples in water, simulated body fluid (SBF), and blood. Several formulations demonstrated significantly higher swelling ratios in all media, notably the samples 1% MgO 0.84 Asp, 2% MgO 0.84 Asp, and 7.5% MgO 0.7:0.3 Asp. These samples exhibited enhanced swelling in water (Figure 4), SBF (Figure 5), and blood (Figure 6) compared to the reference hydrogels. Additionally, 1% MgO 0.84 Glu and 1% MgO 0.7:0.3 Asp showed higher swelling ratios in water and blood, while the 5% MgO 0.3:0.7 Asp and 7.5% MgO 0.3:0.7 Asp samples performed better in SBF and blood.

Several formulations exhibited swelling properties comparable to the reference samples. Specifically, in water, the swelling ratios of 2% MgO 0.84 Asp, 2% MgO 0.5:0.5 Asp, and 2% MgO 0.3:0.7 Asp were similar to those of the reference samples. In SBF, the swelling of 7.5% MgO 0.84 Asp and 1% MgO 0.84 Glu was in line with the reference, while in blood, the 5% MgO 0.7:0.3 Asp sample showed similar swelling behavior to the reference. On the other hand, reduced swelling ratios were observed for the 5% MgO 0.5:0.5 Asp, 7.5% MgO 0.5:0.5 Asp, and 5% MgO 0.7:0.3 Asp samples across all media. Additionally, lower swelling ratios were noted in water and SBF for 5% MgO 0.3:0.7 Asp and 7.5% MgO 0.3:0.7 Asp, in water and blood for 7.5% MgO 0.84 Asp, and in blood only for 2% MgO 0.3:0.7 Asp.

The highest swelling properties were observed for the 1% MgO 0.84 Asp sample in water, the 7.5% MgO 0.3:0.7 Asp sample in SBF, and the 1% MgO 0.7:0.3 Asp sample in blood. Conversely, the poorest swelling properties were recorded for 7.5% MgO 0.84 Asp in both SBF and blood, as well as for 5% MgO 0.5:0.5 Asp in water. Over time, certain samples exhibited a decrease in swelling ratios. In water, the 1% MgO 0.84 Asp sample showed a reduction, while in SBF, the 1% MgO 0.84 Glu and 5% MgO 0.5:0.5 Asp samples exhibited decreased swelling. In blood, the 2% MgO 0.5:0.5 Asp and 5% MgO 0.7:0.3 Asp samples experienced similar reductions. Furthermore, in SBF and blood, the 2% MgO 0.84 Asp, 5% MgO 0.7:0.3 Asp, and 7.5% MgO 0.7:0.3 Asp samples showed a noticeable reduction in swelling ratios. A decrease was also observed for 7.5% MgO 0.84 Asp and 7.5% MgO 0.3:0.7 Asp in water and blood. Conversely, some samples exhibited further swelling over time. The 1% MgO 0.84 Glu and 5% MgO 0.5:0.5 Asp samples swelled further in both water and blood, while the 2% MgO 0.5:0.5 Asp, 7.5% MgO 0.5:0.5 Asp, 5% MgO 0.3:0.7 Asp, and 7.5% MgO 0.84 Asp samples continued to swell in water and SBF. In addition, the 1% MgO 0.7:0.3 Asp, 2% MgO 0.3:0.7 Asp, and 7.5% MgO 0.5:0.5 Asp samples showed continuous swelling across all media. Notably, swelling ratios for the 2% MgO 0.84 Asp sample increased further in water, while 1% MgO 0.84 Asp and 7.5% MgO 0.3:0.7 Asp displayed an increase in SBF.

The swelling properties of chitosan-based hydrogels containing periclase varied significantly across different formulations and testing media. The formulations incorporating 1% MgO 0.84 Asp, 7.5% MgO 0.3:0.7 Asp, and 1% MgO 0.7:0.3 Asp displayed the most favorable swelling characteristics, while formulations with higher concentrations of MgO (5% and 7.5%) and certain Asp ratios demonstrated reduced swelling. Temporal changes in swelling behavior were also observed, with some samples swelling further over time and others exhibiting a decrease. These findings suggest that periclase incorporation and the composition of the Asp/Glutamate ratios influence the swelling properties of chitosan-based hemostatic agents, which may have implications for their performance in different physiological environments.

The swelling behavior of chitosan-based hydrogels containing periclase (MgO) is a crucial factor in determining their potential as hemostatic agents. Hemostasis, the process of stopping bleeding, relies on the ability of materials to rapidly absorb blood, promote clot formation, and provide a physical barrier to blood flow. The swelling properties of these hydrogels, particularly in blood, play a significant role in their effectiveness as hemostatic agents. The hydrogels exhibiting high swelling ratios in blood, such as the 1% MgO 0.84 Asp, 7.5% MgO 0.3:0.7 Asp, and 1% MgO 0.7:0.3 Asp samples, demonstrate an ability to absorb large volumes of liquid. This absorption capacity enables the hydrogels to maintain contact with the wound site, providing a larger surface area for interaction with blood components. Swelling in blood helps to quickly form a physical gel matrix that can effectively trap and concentrate blood cells, such as red blood cells and platelets, facilitating the formation of a blood clot. This localized clot formation is crucial for sealing the wound and preventing further blood loss.

The incorporation of MgO in the hydrogels may further enhance their hemostatic properties. Magnesium ions released from MgO can influence the coagulation cascade, potentially accelerating clot formation. Additionally, the periclase content can help neutralize acidic conditions at the wound site caused by blood acidification, promoting a more favorable environment for coagulation. The ability of these hydrogels to swell and create a gel-like matrix while interacting with blood components aligns with the mechanism of action required for an effective hemostatic agent. Furthermore, the continued swelling observed in certain formulations over time suggests that these hydrogels can adapt to the dynamic nature of wound healing. As the hydrogel swells, it may conform to the wound’s shape, filling gaps and providing additional structural support to the tissue. This sustained interaction with blood and the wound site can enhance the retention of clotting factors and blood cells, ensuring that the hemostatic effect persists as long as necessary. In contrast, hydrogels with lower swelling ratios in blood, such as those with 5% MgO 0.5:0.5 Asp and 7.5% MgO 0.5:0.5 Asp, may not provide the same level of fluid absorption or surface area for clot formation. These formulations may be less effective in rapidly stopping bleeding, as their reduced swelling capacity limits their ability to interact with the wound site and absorb blood effectively.

### 2.3. Chemical Structure Analysis

**Figure 7:**
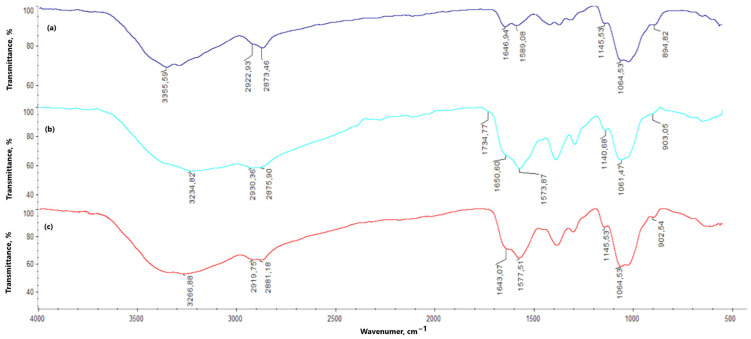
FT-IR spectra of: (**a**)—native chitosan; (**b**)—0.5:0.5 Asp:Glu sample; (**c**)—5% MgO 0.5:0.5 Asp:Glu sample.

In the chitosan FT-IR spectrum (Figure 7), a noticeable band appears at 3355.59 cm^−^^1^, corresponding to the free hydroxy groups present in the chitosan molecule. This band is also observed in the spectra of the hydrogels, both with and without periclase (3100–3300 cm^−^^1^); however, compared to the original chitosan band, it is broader and of lower intensity. The bands related to amino groups appear at 1589.08 and 1145.53 cm^−^^1^, with a marked increase in their intensity for the hydrogel samples (both with and without periclase). The band at 1646.94 cm^−^^1^ corresponds to N-acetylglucosamine. For all hydrogels (with and without periclase) cross-linked with L-aspartic acid, this band shows reduced intensity—1% MgO 0.84 Asp, 2% MgO 0.5:0.5 Asp, 5% MgO 0.5:0.5 Asp, 7.5% MgO 0.5:0.5 Asp, 1% MgO 0.7:0.3 Asp, 5% MgO 0.7:0.3 Asp, 7.5% MgO 0.7:0.3 Asp, 5% MgO 0.3:0.7 Asp, 7.5% MgO 0.3:0.7 Asp—or is not observed in the spectrum—2% MgO 0.84 Asp, 7.5% MgO 0.84 Asp. A different result is observed with L-glutamic acid as the primary cross-linking agent, where an increase in band intensity is noted relative to the reference sample and chitosan—1% MgO 0.84 Glu. When analyzing the hydrogels in terms of periclase concentration, a decrease in the intensity of this band is also observed. Bonds between -CH- and -CH_2_- groups correspond to values of 2922.93 and 2873.46 cm^−^^1^, while β-glycosidic bonds between chitosan mer units appear at 1064.53 cm^−^^1^ (1060–1070 cm^−^^1^). The band at 894.82 cm^−^^1^ is attributed to glucopyranose rings. In the spectra of hydrogels, with and without periclase, these bands show no shifts. However, bands corresponding to the bonds between -CH- and -CH_2_- groups (2800 to 3000 cm^−^^1^) and β-glycosidic bonds (1060–1070 cm^−^^1^) do exhibit shifts. In some samples without periclase (0.5:0.5 Asp, 0.7:0.3 Asp, and 0.3:0.7 Asp), bands are also noted at 1734.77, 1725.77, and 1727.93 cm^−^^1^, corresponding to ester bonds formed between hydroxy groups in chitosan and carboxylic groups from amino acids. Below are the spectra of chitosan and samples with and without periclase.

The chemical crosslinking mechanism between chitosan and amino acids containing two carboxylic groups, such as aspartic acid and glutamic acid, involves the formation of amide bonds through a condensation reaction between the amine groups (-NH_2_) of chitosan and the carboxylic groups (-COOH) of the amino acids. Under microwave-assisted conditions, this process is significantly enhanced due to rapid heating and increased molecular mobility, which facilitates efficient bond formation.

The reaction begins with the thermal activation of the carboxylic groups, leading to their conversion into more reactive intermediates, such as anhydrides or acyl species. This activation promotes a nucleophilic attack by the primary amine groups of chitosan, resulting in the formation of covalent amide (-CONH-) bonds that link the amino acids to the chitosan backbone. Given that both aspartic acid and glutamic acid contain two carboxylic groups, they can react with multiple chitosan chains, creating a crosslinked polymer network. This crosslinking process enhances the structural integrity and stability of the hydrogel.

**Figure 8:**
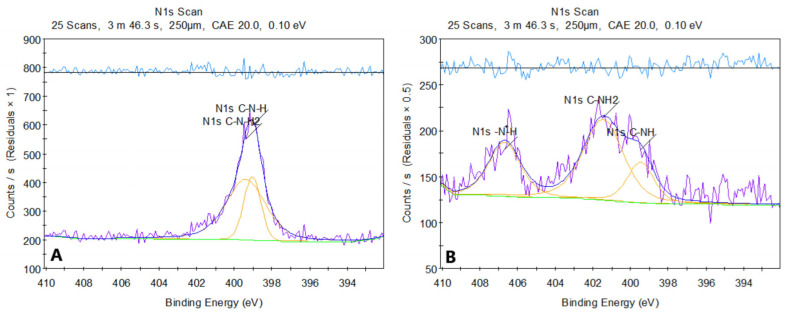
XPS spectra of (**A**) native chitosan and (**B**) crosslinked chitosan.

Figure 8 presents the XPS spectrum of pure chitosan (a) and sample 2. The XPS spectrum of chitosan before modification shows two peaks in the N 1s region, located around ~399 eV. The first corresponds to the amine group C-NH_2_ (~399 eV), and the second to the amide group C-NH (~398.8–399.5 eV). Both signals are characteristic of the structures present in chitosan, where nitrogen exists in the form of amine and amide groups, without significant protonation or other strong chemical modifications.

After modification of chitosan with asparagine acid, the N 1s spectrum shows a shift in the peaks towards higher binding energies. The peak around ~400 eV (C-NH) has shifted compared to the original spectrum, which may suggest the formation of amide bonds (-CONH-) between chitosan and aspartic acid. Additionally, a new peak around ~401 eV (R-NH_3_^+^) appeared, indicating protonation of amine groups due to the reaction in an acidic environment. The new feature in the ~406–407 eV range is particularly noteworthy and may arise from protonated amides (CONH_2_^+^), suggesting that some of the amide or amine groups underwent modification, likely due to interactions with dicarboxylic acid.

The changes in the spectrum after modification indicate the introduction of new functional groups, such as amides, and protonation of amine groups. The analysis of this spectrum suggests that the chemical modification of chitosan with aspartic acid leads to the formation of new amide bonds and changes in the chemical environment of nitrogen.

**Figure 9:**
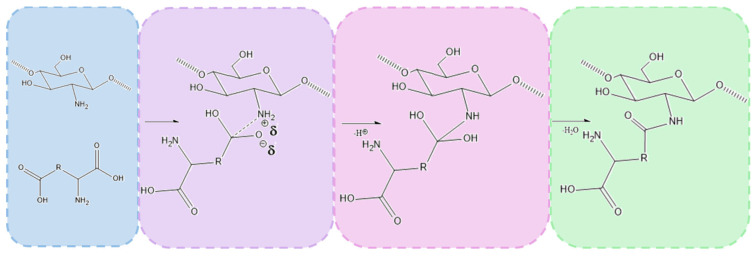
Proposed crosslinking mechanism.

The successful formation of amide bonds is confirmed by both FT-IR and XPS analyses. FT-IR spectra reveal the appearance of characteristic amide I (C=O stretching at ~1640–1660 cm^−^^1^) and amide II (N-H bending at ~1550 cm^−^^1^) bands. XPS analysis further supports the crosslinking mechanism by detecting a new N 1s peak (~399–401 eV), corresponding to amide nitrogen, along with shifts in the O 1s and C 1s spectra, indicative of newly formed amide linkages. Overall, microwave-assisted crosslinking between chitosan and dicarboxylic amino acids results in the formation of a stable, covalently bonded network that enhances the mechanical properties, hemostatic potential, and antibacterial activity of the hydrogel. Proposed mechanism is given in Figure 9.

### 2.4. Morphology Study

**Figure 10:**
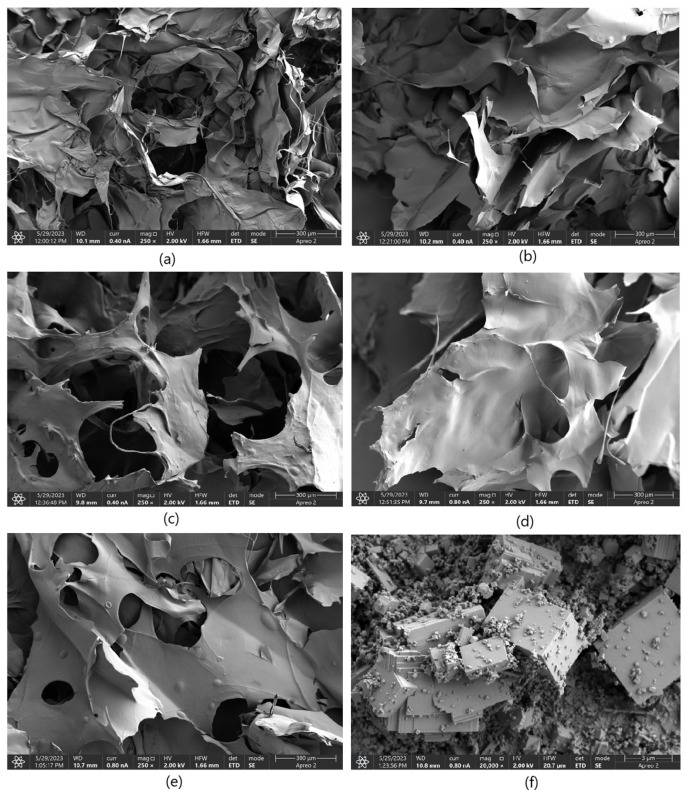
SEM images of the samples: (**a**)—1% MgO 0.84 Glu; (**b**)—7.5% MgO 0.7:0.3 Asp:Glu; (**c**)—5% MgO 0.3:0.7 Asp:Glu; (**d**)—2% MgO 0.84 Asp; (**e**)—2% MgO 0.5:0.5 Asp:Glu; (**f**)—periclase nanoparticles.

**Figure 11:**
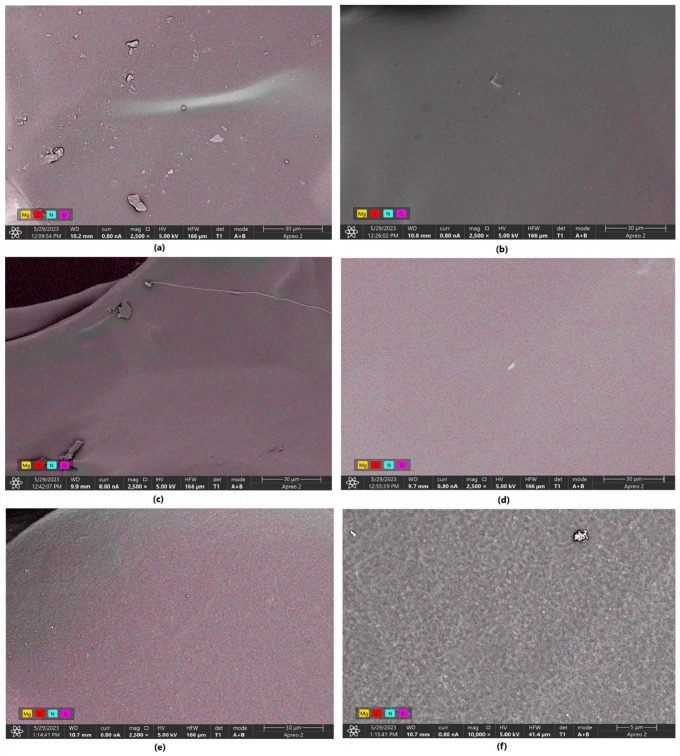
EDS mapping of the samples: (**a**)—1% MgO 0.84 Glu; (**b**)—1% MgO 0.84 Asp; (**c**)—5% MgO 0.3:0.7 Asp:Glu; (**d**)—2% MgO 0.84 Asp; (**e**)—2% MgO 0.5:0.5 Asp:Glu; (**f**)—7.5% MgO 0.7:0.3 Asp:Glu.

In the SEM images (Figure 10) of the samples, numerous pores are observed. The samples exhibit an open-pore structure, which facilitates the infiltration of hydrophilic molecules into the matrix, as well as cellular adhesion, migration, and proliferation. This structure also allows access to nutrients, enables waste product removal, and supports gas exchange. In the hydrogels where L-glutamic acid served as the primary cross-linking agent (1% MgO, 0.84 Glu, and 7.5% MgO, 0.3:0.7 Asp), the surface was distinctly rougher compared to samples with L-aspartic acid as the main agent or an equal contribution of both amino acids—1% MgO 0.84 Asp, 5% MgO 0.5:0.5 Asp, and 1% MgO 0.7:0.3 Asp. Elemental mapping (Figure 11 and Table 1, Table 2, Table 3, Table 4 and Table 5) results confirm the presence of periclase particles on the hydrogel surface, showing magnesium and oxygen (the latter also being a component of chitosan and amino acids, alongside carbon and nitrogen). The percentage of magnesium relative to other elements is minimal—for all samples except 7.5% MgO 0.3:0.7 Asp, it is 0.1%, while for this latter sample, it is 0.2%. Furthermore, point mapping of the surface of sample 7.5% MgO 0.3:0.7 Asp (percentage composition for points 1 and 4) also confirms this (40.9% and 43.4% magnesium, 46.6% and 46.8% oxygen). A comparison of SEM images for the 7.5% MgO 0.3:0.7 sample with SEM images of pure periclase further supports the presence of magnesium oxide crystals on the sample surfaces. In the surface composition chart, in addition to peaks for carbon, oxygen, nitrogen, and magnesium, a peak for gold is also visible (used as a coating with a layer thickness of 6 nm), appearing at around 2.2 keV. Below are SEM images showing the structures of individual samples and pure periclase.

### 2.5. Porosity and Density Study

**Figure 12:**
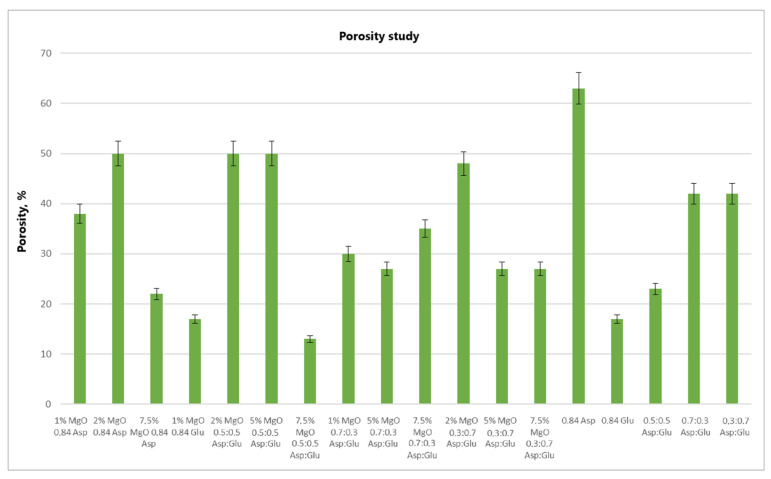
Volume porosity study.

**Figure 13:**
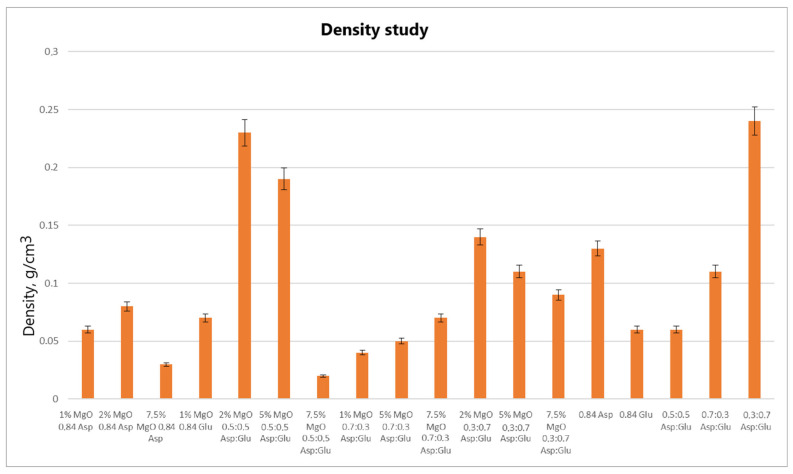
Volume density study.

Analyzing the volume porosity (Figure 12) and volume density (Figure 13) values of the obtained samples reveals that for 2% MgO 0.5:0.5 Asp and 5% MgO 0.5:0.5 Asp, both values are higher compared to the reference hydrogels (without periclase). Increased porosity was also observed for 2% MgO 0.3:0.7 Asp, while higher density was noted for 2% MgO 0.84 Asp and 1% MgO 0.84 Glu. Conversely, lower values for both porosity and density were obtained for 7.5% MgO 0.84 Asp, 7.5% MgO 0.5:0.5 Asp, 1% MgO 0.7:0.3 Asp, 5% MgO 0.7:0.3 Asp, and 7.5% MgO 0.7:0.3 Asp. Lower porosity was also observed for 1% MgO 0.84 Asp, 5% MgO 0.3:0.7, and 7.5% MgO 0.3:0.7 Asp. The same porosity value was obtained for the 1% MgO 0.84 Glu sample, with a similar value observed for 2% MgO 0.84 Asp, 2% MgO 0.3:0.7 Asp, 5% MgO 0.3:0.7 Asp, and 7.5% MgO 0.3:0.7 Asp. The sample with the highest porosity and density was 2% MgO 0.5:0.5 Asp, while the lowest values were found in 7.5% MgO 0.5:0.5 Asp. Samples 2% MgO 0.84 Asp and 2% MgO 0.5:0.5 Asp also exhibited similar porosity.

### 2.6. Biodegradation Study

**Figure 14:**
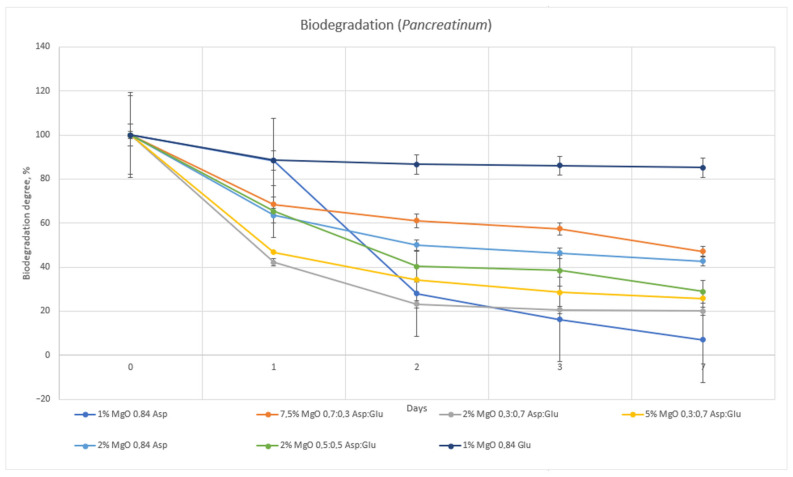
Biodegradation carried out in SBF solution containing pancreatinum.

**Figure 15:**
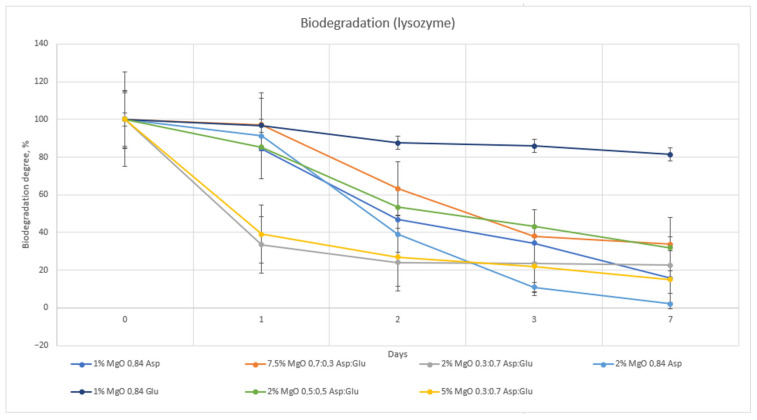
Biodegradation carried out in SBF solution containing lysozyme.

The hydrogel sample 1% MgO 0.84 Glu was the least susceptible to biodegradation by both lysozyme and Creon. The samples with the highest biodegradation rates were 1% MgO 0.84 Asp in Creon and 2% MgO 0.84 Asp in lysozyme. Comparing the two enzymes, lysozyme exhibited superior degradative properties (Figure 14 and Figure 15).

### 2.7. Cytototxicity Study

**Figure 16:**
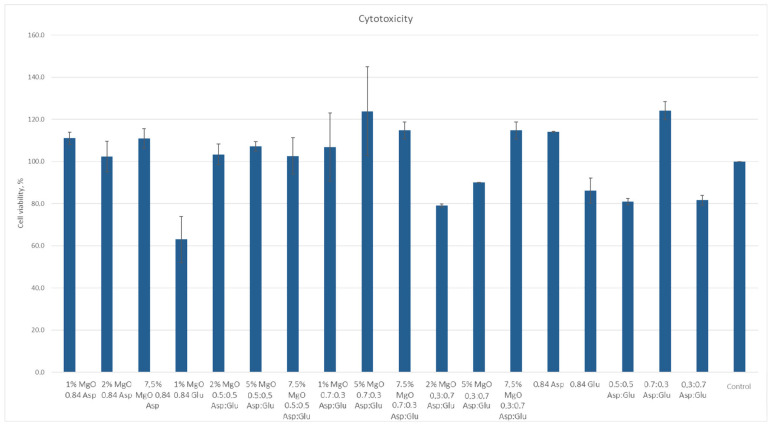
XTT assay results.

**Figure 17:**
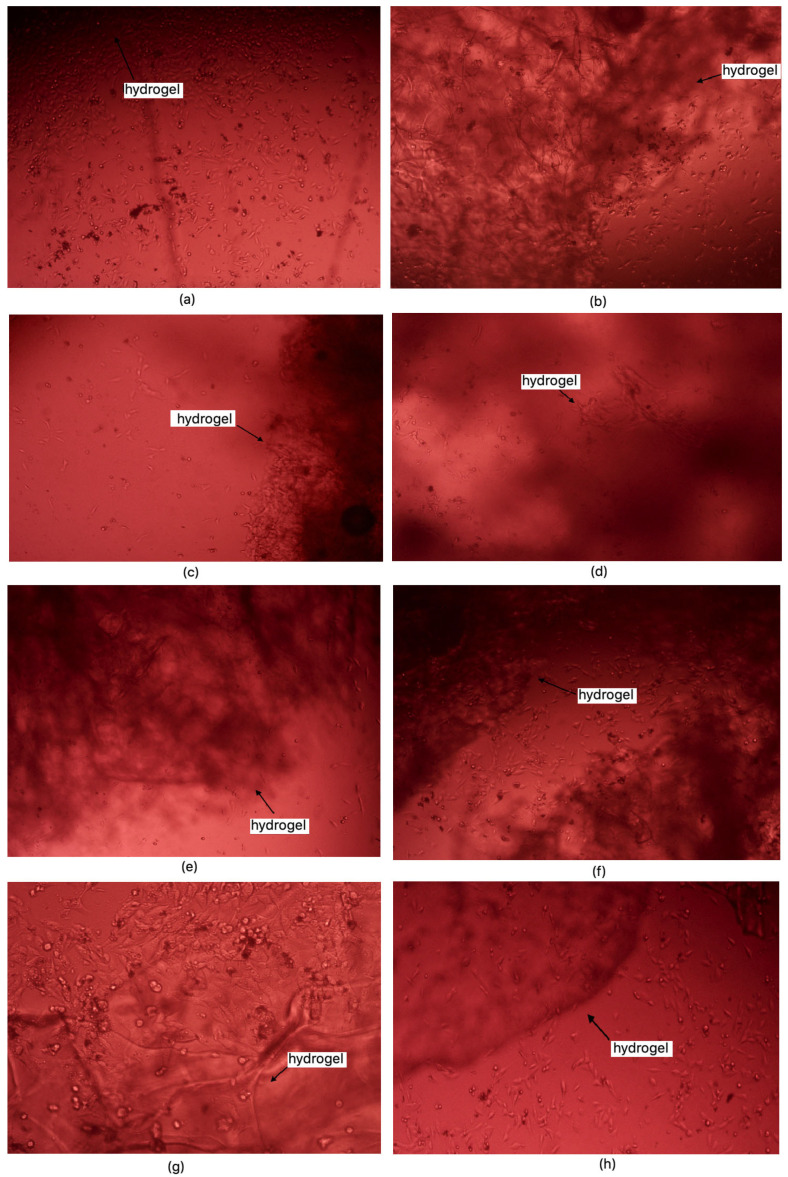
Cell morphology after direct contact with tested samples for 48h: (**a**)—Sample 1% MgO 0.84 Asp; (**b**)—Sample 7.5% MgO 0.84 Asp (**c**)—Sample 2% MgO 0.84 Asp (**d**)—Sample 0.3 Asp:0.7 Glu (**e**)—Sample 2% MgO 0.84 Glu (**f**)—Sample 7.5% MgO 0.5 Asp:0.5 Glu (**g**)—Sample 7.5% MgO 0.5 Asp:0.5 Glu (**h**)—Sample 7.5% MgO 0.3 Asp:0.7 Glu.

According to ISO 10933 [37] standards for medical devices, a material is considered non-cytotoxic if cell viability is at least 70% relative to the control sample. Among the samples studied, all except 1% MgO 0.84 Glu were non-cytotoxic. The sample with the lowest cytotoxicity was 5% MgO 0.7:0.3 Asp, while the highest cytotoxicity (excluding 1% MgO 0.84 Glu) was observed in 2% MgO 0.3:0.7 Asp. Increased cell proliferation relative to the control was observed in samples of 1% MgO 0.84 Asp, 2% MgO 0.84 Asp, 7.5% MgO 0.84 Glu, 2% MgO 0.5:0.5 Asp, 5% MgO 0.5:0.5 Asp, 7.5% MgO 0.5:0.5 Asp, 1% MgO 0.7:0.3 Asp, 5% MgO 0.7:0.3 Asp, 7.5% MgO 0.7:0.3 Asp, and 7.5% MgO 0.3:0.7 Asp. Greater cell survival in extracts of biomaterials containing periclase, compared to samples without it, was observed for 2% MgO 0.5:0.5 Asp, 5% MgO 0.5:0.5 Asp, 7.5% MgO 0.5:0.5 Asp, 5% MgO 0.3:0.7 Asp, and 7.5% MgO 0.3:0.7 Asp. Microscopic images show increased proliferation of L929 mouse fibroblasts on most hydrogel structures (except 1% MgO 0.84 Glu). A greater number of cells over time, comparing 24 to 48 h after the start of incubation, is also evident. Most cells display normal morphology, with an elongated shape and flat adherence to the surface of the plate. Dead cells, appearing dark and forming aggregates, are also visible in the microscopic images (Figure 16 and Figure 17).

**Figure 18:**
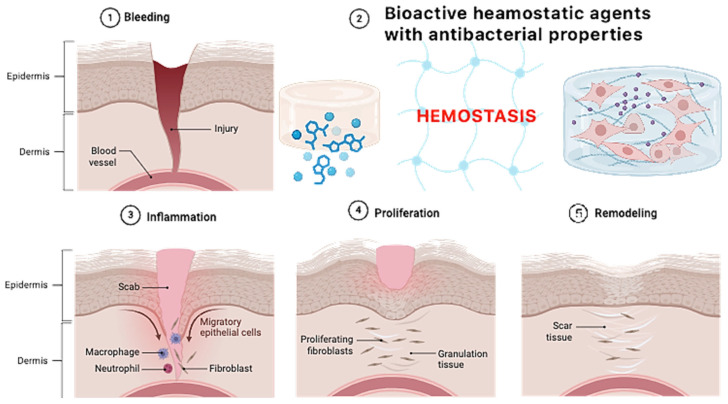
Possible future applications.

Figure 18 reveals possible future application of newly developed biomaterials. Both FT-IR and XPS analyses provided clear evidence of the successful crosslinking of chitosan with dicarboxylic amino acids—namely aspartic acid, glutamic acid, and their combination—resulting in the formation of amide bonds between the amino groups of chitosan and the carboxyl groups of the amino acids. This modification was crucial in enhancing the hemostatic properties of the material while maintaining the biological activity of chitosan. The introduction of amino acids as crosslinkers presents significant advantages over conventional chemical crosslinkers such as glutaraldehyde or genipin, which tend to drastically reduce the availability of amino groups due to excessive crosslinking. Chitosan’s hemostatic efficiency is largely attributed to its cationic nature, which stems from its free amino groups. However, when crosslinked with traditional agents, these groups are significantly diminished, leading to reduced biological activity. In contrast, the incorporation of amino acids as crosslinking agents helps to preserve the essential amino functionalities within the hydrogel network. Although amide bonds form between the amino groups of chitosan and the carboxyl groups of aspartic and glutamic acids, the final hydrogel structure retains free amino groups from the amino acids themselves. This is a crucial aspect, as these amino groups contribute to the hemostatic potential by maintaining interactions with negatively charged components of blood, promoting platelet adhesion and activation. Furthermore, the protonation of the amino groups in the amino acids at different pH values significantly broadens the biomedical applicability of the developed hydrogels. Unlike the amino groups in chitosan, which predominantly exist in a protonated NH_3_^+^ state at acidic pH, the amino groups in the incorporated amino acids display pH-dependent protonation behavior. This property enhances the material’s functionality in varying physiological environments, ensuring effective hemostatic action across a broader range of conditions. Another advantage of this approach is the biocompatibility of the employed crosslinking agents. While glutaraldehyde and genipin are known to induce cytotoxicity due to the formation of highly reactive aldehyde or secondary crosslinking structures, the use of naturally occurring amino acids minimizes potential adverse biological effects. This makes the amino acid-crosslinked chitosan hydrogels more suitable for clinical applications, where minimizing immune responses and cytotoxicity is of paramount importance. Overall, the results indicate that chemical crosslinking with dicarboxylic amino acids is an effective strategy for enhancing the structural and hemostatic properties of chitosan-based hydrogels while preserving their biological activity.

### 2.8. Antibacterial Properties Study

The performed study demonstrated a notable inhibitory effect on the growth of both *Staphylococcus aureus* and *Escherichia coli*. Colony counting for *S. aureus* was not conducted due to the density and merging of colonies. However, results for the higher dilution of samples were recorded and summarized in Table 6, Table 7, Table 8 and Table 9. Among the various hydrogel formulations, the sample 1% MgO 0.84 Glu exhibited the strongest antibacterial properties against both bacterial strains. However, given its cytotoxicity, the second-best antibacterial hydrogels were identified as 7.5% MgO 0.7:0.3 Asp for *E. coli* and 5% MgO 0.3:0.7 Asp for *S. aureus*. Nevertheless, all the hydrogels demonstrated inhibitory effects on the growth and proliferation of both bacterial strains, with a clear reduction in colony count observed 2 h and 24 h post-infection of the biomaterials.

The observed antibacterial properties align with existing knowledge regarding chitosan’s antimicrobial activity, which is primarily attributed to the protonable NH_2_ groups present on its molecular structure. These cationic groups facilitate electrostatic interactions with the negatively charged bacterial cell membranes, leading to membrane disruption and subsequent leakage of intracellular contents. Chitosan’s antimicrobial effect is multifaceted: chitosan of lower molecular mass can penetrate bacterial cells and interact with negatively charged genetic material, such as DNA, while chitosan with higher molecular mass tends to interact with the cell wall and membrane components, blocking both passive and active nutrient transport. Furthermore, chitosan can chelate crucial ions like Mg^2^^+^ and Ca^2^^+^, disrupting essential cellular processes.

**Figure 19:**
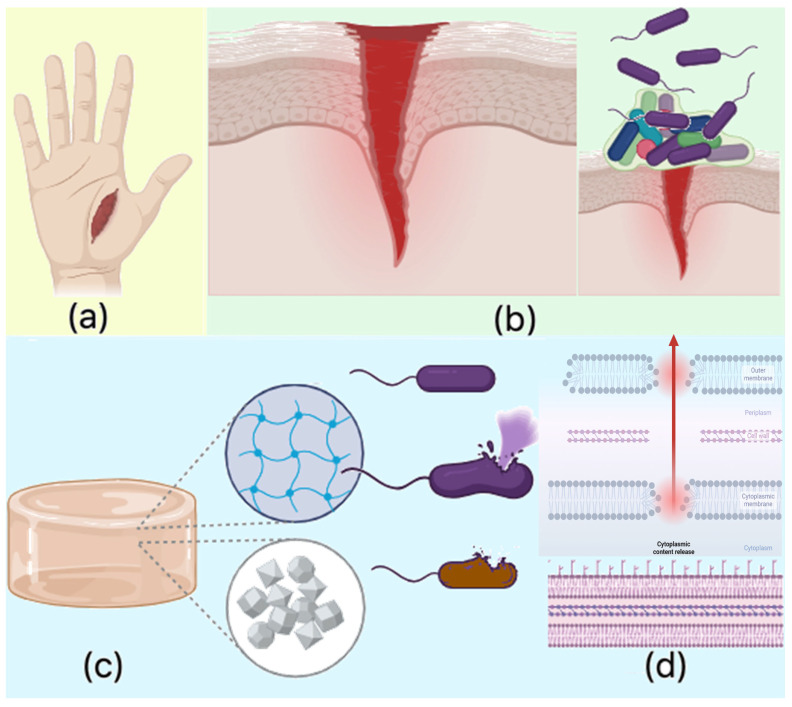
Possible mode of action of hemostatic agents: (**a**)—injury; (**b**)—bleeding, wound formation, and biofilm formation; (**c**)—antibacterial activity of the biomaterial; (**d**)—pathogen membrane disruption and bacteria death.

In this study, the antibacterial activity of the chitosan-based hydrogels was further enhanced by the incorporation of periclase nanoparticles (MgO). The release of magnesium ions (Mg^2^^+^) from MgO contributes to membrane destabilization, impeding bacterial metabolic functions and ultimately leading to cell death. Additionally, the magnesium ions may induce oxidative stress by generating reactive oxygen species (ROS), which are harmful to bacterial cells. This oxidative damage further disrupts bacterial cell functioning, contributing to the hydrogels’ antibacterial properties. The crystalline nature of the MgO nanoparticles may also interfere with biofilm formation, a critical factor in bacterial resistance, by preventing the attachment and accumulation of bacterial cells at the wound site. Additionally, the hydrogel’s ability to swell in biological fluids such as simulated body fluid (SBF) and blood increases its surface area, enhancing its interaction with bacterial cells. This physical property, combined with the chemical antibacterial effects of chitosan and MgO, prevents bacterial adhesion and proliferation, significantly reducing the risk of infection at the wound site. The combination of chitosan and MgO in the hydrogel matrix results in a potent antibacterial effect against both *S. aureus* and *E. coli*, with enhanced activity observed against *E. coli*. The antibacterial properties are attributed to several mechanisms, including the disruption of bacterial cell membranes by chitosan, ion chelation, and the generation of oxidative stress by magnesium ions. Moreover, the crystalline nature of MgO nanoparticles contributes to the inhibition of biofilm formation. These properties make the chitosan-based MgO hydrogels highly effective in preventing bacterial infection, establishing them as promising candidates for use as hemostatic agents in wound care applications (Figure 19).

The number of colony-forming units (CFU) of *Escherichia coli* is summarized in Table 6.

After accounting for the dilutions and the volume of bacterial suspension taken for swabbing.

The number of colony-forming units (CFU) of *Staphylococcus aureus* is summarized in Table 8.

## 3. Materials and Methods

### 3.1. Materials

Fungal chitosan, sourced from *Aspergillus niger*, was used in this study. It is a powder with a color range from white to light yellow or light brown. The chitosan is soluble in 1% acetic acid at a concentration of 1%, forming a clear or slightly turbid solution that is colorless or light yellow-brown. At a 1% concentration in 1% acetic acid and at 20 °C, the chitosan exhibits a viscosity ranging from 10 to 120 cps. The material has an ash content of ≤2.0% and a moisture loss upon drying of ≤15.0%. Additionally, it meets stringent quality control standards for heavy metals, with arsenic (As) levels ≤ 2.0 ppm, lead (Pb) ≤ 1.0 ppm, cadmium (Cd) ≤ 0.5 ppm, mercury (Hg) ≤ 0.5 ppm, and total heavy metals ≤ 20 ppm. The chitosan has a degree of deacetylation (DA) of ≥85.0%, ensuring its high biocompatibility and suitability for various applications. Glucose Broth Agar, *E. coli* ATCC^®^ 43888 ATCC^®^ BAA-2312^TM^, and *S. aureus* strains were purchased from PolAura, Dywity, Poland. For crosslinking, *L*-aspartic acid, *L*-glutamic acid, propylene glycol, 2-propanol, and ethanol 96% were obtained from Sigma Aldrich, (Poznań Poland). Periclase was obtained under 1300 °C via calcination of MgO under inert gas. Antibiotics, antibiotics/antimycotics, DMEM cell culture medium with and without phenol red, trypsin, FBS, L929 cell line (European Cell Culture Collection ECCC), lyophilized blood, and human lysozyme were purchased from Sigma Aldrich. The XTT assay was purchased from Roche, Sigma Aldrich, (Poznań, Poland). Kreon Travix (pancreatic enzymes) was purchased at the DrMax pharmacy (Abbott Products GMBH, Wiesbaden, Germany).

### 3.2. Methods

#### 3.2.1. Periclase Synthesis

Magnesium oxide (MgO) was synthesized from reagent-grade magnesium hydroxide (Mg(OH)_2_) via thermal dehydration at temperatures above 300 °C, leading to complete conversion to MgO.

Thermal treatment was conducted in a temperature range of 500 °C to 1200 °C, with 100 °C increments. Each sample was calcined for 4 h. Approximately 10–15 g of magnesium hydroxide powder was placed in a quartz crucible, ensuring that the powder thickness did not exceed 5 mm to facilitate uniform heating and prevent ejection due to rapid gas release during dehydration. The crucibles containing Mg(OH)_2_ samples were placed in a furnace pre-cooled to below 100 °C and then heated to the target temperature. Pre-heating the furnace or using thick powder layers was avoided to minimize disturbances caused by rapid dehydration. After reaching the desired temperature and maintaining it for the designated time, the samples were cooled inside the furnace to approximately 200 °C and then transferred to a desiccator. Once cooled to ambient temperature, the samples were stored in airtight containers. MgO obtained at temperatures below 900 °C exhibited high reactivity and readily rehydrated in the presence of atmospheric moisture to reform magnesium hydroxide. Conversely, MgO synthesized above 900 °C demonstrated low reactivity and a high degree of crystallinity, resembling the periclase crystalline structure. This characteristic makes high-temperature MgO suitable for the production of refractory materials, which exhibit excellent chemical resistance and a high melting point exceeding 2800 °C. To confirm the formation of highly crystalline MgO, the obtained powders were subjected to X-ray diffraction (XRD) analysis and transmittance electron microscopy (TEM).

#### 3.2.2. Biomaterials Synthesis

Magnesium oxide (MgO) was obtained by dehydrating magnesium hydroxide (Mg(OH)_2_) at 500–1200 °C for 4 h per batch. To prevent material loss, Mg(OH)_2_ was placed in a quartz crucible with a ≤5 mm layer and gradually heated. MgO obtained below 900 °C remained reactive, while above 900 °C, it became highly crystalline, resembling periclase, making it suitable for refractory materials due to its high chemical resistance and >2800 °C melting point.

XRD analysis confirmed increasing crystallinity with temperature, as indicated by the rising intensity of MgO peaks. The highest peak intensity was observed for >1000 °C samples, confirming the formation of highly crystalline periclase-phase MgO.

In a 250 mL beaker, an appropriate amount of amino acid was dissolved in 22 mL of distilled water (25 mL for samples without added magnesium oxide) at 120 °C on a magnetic stirrer set to 300 revolutions per minute, with the beaker covered by a watch glass. Then, 0.5 g of chitosan was added. Meanwhile, a specified amount of periclase (magnesium oxide) was suspended in 3 mL of distilled water using an ultrasonic bath. The magnesium oxide suspension was then added to the beaker contents and stirred until a homogeneous mixture was obtained. Next, 10 mL of propylene glycol was added, and the reaction vessel was left on the magnetic stirrer until a uniform suspension was achieved. The beakers were then placed in a microwave oven and subjected to microwave radiation at 800 W until a color change (light orange, light brown) was observed. This color change likely indicates chitosan oxidation rather than a specific periclase-induced reaction and should be clarified in future studies. After synthesis, the resulting hydrogels were rinsed with 200 mL of distilled water, and the filtrate was collected for elemental analysis of magnesium (periclase) content. The rinsing steps were repeated to allow the hydrogels to swell, and the samples were rinsed until a pH of 7 was achieved, ensuring that the solution pH was neutralized. Table 10 summarizes the quantities of reagents required to obtain the samples (listing only hydrogels that were successfully synthesized).

The purified samples were dried on sieves and paper towels, then placed in containers to prevent contamination and stored in a freezer. Once frozen, the samples were transferred to a lyophilizer for complete drying. To carry out comparisons of the newly developed biomaterials with standard antibiotics, control series of samples had been prepared as follows. Dried samples were weighed and then swollen using an ethanol solution of penicillin-streptomycin. A volume of a solution containing an amount of drug equal to 0.1% of the sample’s mass was used for incorporation. The hydrogels were left to dry completely overnight.

#### 3.2.3. Swelling Ratio Determination

Ready samples, including antibiotic-covered hydrogel pieces, were weighed and then swollen in 1.5 mL of water, simulated body fluid (SBF), and human blood (samples were immersed in 3 mL of bodily fluid). Each test was performed in triplicate. After 10 and 60 min of immersion, the hydrogels were weighed. The swelling ratio was calculated using the following formula:

(1) $$S_{D}=\frac{W_{S}}{W_{D}}$$

where

$S_{D}$—swelling degree
$W_{D}$—dried hydrogel weight [g]
$W_{S}$—swollen hydrogel weight after 10 and 60 min after immersion

#### 3.2.4. FT-IR and XPS Chemical Structure Analysis

Dried hydrogel and chitosan samples were placed on a monolithic diamond crystal. The materials were pressed onto the surface using an ATR adapter. FT-IR spectra were recorded, processed, and analyzed to assess the chemical structure. For the experiments, a Thermo Nicolet Nexus 470 FT-IR spectrophotometer (Thermo Fisher Scientific, Waltham, MA, USA) was used with a scan range from 4000 cm^−^^1^ to 400 cm^−^^1^, resolution 4^−^^1^, 32 scans per analysis. The chemical composition of the surface was analyzed using X-ray photoelectron spectroscopy (XPS). Prior to measurement, the sample was placed on a dedicated sample holder. The samples did not require any special preparation. The radiation source was an Al Kα lamp (1486.6 eV) with a power of 360 W. Measurements were conducted in a vacuum range of 10^−9^ to 10^−8^ mbar. Survey spectra were recorded over a wide energy range from 1350 eV to 0 eV with a step size of 1.0 eV at a pass energy (CAE) of 150 eV (5 scans). High-resolution spectra for selected elements were recorded in their characteristic binding energy ranges with a step size of 0.1 eV at a pass energy (CAE) of 25 eV. Each sample was scanned 20 times. Survey spectra were recorded for all analyzed samples, while detailed spectra for nitrogen (N 1s), oxygen (O 1s), and carbon (C 1s) were specifically obtained. Deconvolution of high-resolution spectra allowed the assignment of specific chemical states of elements to the observed components. XPS spectra were analyzed using Avantage software (version 5.975) from Thermo Fisher Scientific. Additionally, publicly available databases and the Handbook of X-ray Photoelectron Spectroscopy were used.

#### 3.2.5. Morphology Analysis via SEM and TEM

Selected samples, based on previous results, were subjected to SEM morphology analysis. Prior to examination, dried hydrogels were coated with a 6 nm layer of gold. The structure was observed under a scanning electron microscope, and elemental mapping was performed via EDS. Elemental point mapping was additionally conducted on the hydrogel cross-linked with 0.3 g of L-aspartic acid, 0.7 g of L-glutamic acid, and containing 7.5% periclase relative to the chitosan and amino acid mass. Imaging was conducted at magnifications of 150×, 250×, 500×, 8000×, and for some samples, at higher magnifications (2500×, 16,000×, 30,000×). MgO nanoparticles were analyzed using a Transmission Electron Microscope (TEM) from JEOL (Peabody, MA, USA). The nanoparticles were dispersed in ethanol to prevent agglomeration, and a drop of the suspension was deposited onto a copper grid.

#### 3.2.6. Volume Porosity and Volume Density Determination

Between 5.5 and 8.5 mL of isopropanol was added to a 10 mL graduated cylinder. Samples were weighed before immersion in isopropanol. Three measurements of liquid height were taken: before immersion, immediately after sample immersion, and after removing the sample 5 min later. This procedure was repeated for each sample. Density and porosity were calculated as follows:

(2) $$d=\frac{W}{V_{2}-V_{3}}$$

where

***W***—weight, g
***d*** = density [g/cm^3^],
***V***_2_ = volume of isopropanol with the sample [cm^3^],
***V***_3_ = volume of isopropanol after sample removal [cm^3^].

(3) $$p=\frac{V_{1}-V_{3}}{V_{2}-V_{3}}*100\%$$

where

***p*** = porosity (%),
***V***_1_ = initial isopropanol volume [cm^3^],
***V***_2_ = volume of isopropanol with sample [cm^3^],
***V***_3_ = volume of isopropanol after sample removal [cm^3^].

#### 3.2.7. Biodegradation Study

Solutions of Creon (active ingredients: 10,000 Ph.Eur. lipase units, 8000 Ph.Eur. amylase units, 600 Ph.Eur. protease units) and human lysozyme in sterile PBS (concentrations of 20 mg/L and 10 mg/L, respectively) were prepared. Samples were weighed and placed in enzyme solutions within baskets and incubated at 37 °C. Every 24 h, samples were removed, dried, and weighed. Testing was conducted over 7 days in triplicate. The biodegradation rate was calculated as follows:

(4) $$BD=\frac{W_{0}-W_{t}}{W_{0}}*100\%$$

where

***BD*** = biodegradation rate [%],
***W***_0_ = initial sample weight [g],
***W_t_*** = sample weight at a specific time [g].

#### 3.2.8. Cytotoxicity Evaluation

Hydrogel samples were weighed and sterilized using UV radiation. Each sample was placed in a well containing 1 mL of L929 mouse fibroblast cell suspension and 2 mL of culture medium. Control samples were also prepared. Testing was conducted in triplicate for each sample and control. Plates were incubated, and after 24 and 48 h, cell morphology and structure were observed at 40× and 100× magnification. On the seventh day, an XTT assay was performed. Cytotoxicity for each sample was calculated based on absorbance at 450 nm.

#### 3.2.9. Antibacterial Properties Testing

Initial bacterial cultures (10 mL) of Escherichia coli and Staphylococcus aureus were prepared, with dilution series of 10^−^^1^, 10^−^^2^, and 10^−^^3^. The initial concentration of 105 colony-forming units per milliliter (CFU/mL) Selected samples based on prior test results were weighed and placed in multi-well plates. Controls were also prepared. For each sample, 1 mL of bacterial suspension at 10^−^^1^ and 10^−^^3^ dilution was added. After 2, 4, 6, and 24 h, 50 μL of the suspension was taken, plated on solid agar-glucose plates, and incubated at 37 °C for 24 h.

## 4. Conclusions

This study provides insights into the cross-linking and structural characteristics of chitosan-based hydrogels modified with periclase. Successful preparation of highly crystalline and stable MgO was confirmed by XRD, SEM, and TEM methods. FT-IR and XPS analysis showed that cross-linking occurs through chitosan’s free amino groups, with an increase in amino group intensity and a reduction in *N*-acetyl-D-glucosamine. Despite cross-linking, the modified chitosan retained more amino groups. SEM analysis confirmed a porous structure, and magnesium presence was verified through atomic spectroscopy and surface mapping, with significant magnesium content observed at 7.5% periclase concentration. However, no clear correlation was found between periclase concentration and key hydrogel properties, such as swelling ratio, density, biodegradation, or antibacterial efficacy. The hydrogels exhibited inherent antibacterial properties, indicating potential biomedical applications.

XPS analysis further confirmed magnesium-chitosan interactions, showing shifts in nitrogen and oxygen binding energies, supporting the FT-IR and SEM findings. Despite no significant improvements in mechanical properties, the hydrogels’ antibacterial activity and porous structure suggest promise for tissue engineering and drug delivery. Further optimization of hydrogel composition is needed to enhance performance for specific biomedical uses.

## Figures and Tables

**Table 1:** Quantitative EDS of a 1% MgO 0.84 Glu sample.

Element	Atomic %	Atomic % Error	Weight %	Weight % Error	Net Counts
C	36.5	0.2	30.5	0.2	70,620
N	8.4	0.8	8.2	0.7	4915
O	54.9	0.5	61.1	0.6	47,554
Mg	0.1	0.1	0.2	0.1	173

**Table 2:** Quantitative EDS of a 1% MgO 0.84 Asp sample.

Element	Atomic %	Atomic % Error	Weight %	Weight % Error	Net Counts
C	41.4	0.3	35.0	0.3	15,932
N	7.2	1.1	7.1	1.1	793
O	51.3	0.9	57.7	1.0	8589
Mg	0.1	0.1	0.1	0.1	16

**Table 3:** Quantitative EDS of 5% MgO 0.3:0.7 Asp:Glu.

Element	Atomic %	Atomic % Error	Weight %	Weight % Error	Net Counts
C	32.9	0.2	27.2	0.2	77,009
N	8.6	0.7	8.3	0.6	6359
O	58.4	0.5	64.4	0.5	62,838
Mg	0.1	0.1	0.1	0.1	131

**Table 4:** Quantitative EDS of 2% MgO 0.84 Asp.

Element	Atomic %	Atomic % Error	Weight %	Weight % Error	Net Counts
C	32.3	0.2	26.6	0.2	60,407
N	7.8	0.7	7.5	0.7	4627
O	59.8	0.5	65.7	0.6	52,056
Mg	0.1	0.1	0.1	0.1	96

**Table 5:** Quantitative EDS of 2% MgO 0.5:0.5 Asp:Glu.

Element	Atomic %	Atomic % Error	Weight %	Weight % Error	Net Counts
C	36.0	0.2	29.9	0.2	83,086
N	6.9	0.7	6.7	0.7	4871
O	56.9	0.5	63.0	0.5	60,060
Mg	0.2	0.1	0.3	0.1	321

**Table 6:** Results of the antibacterial properties of the samples against Escherichia coli.

Sample	2 h	4 h	6 h	24 h
1% MgO 0.84 Glu	480	390	121	61
7.5% MgO 0.7:0.3 Asp:Glu	320	216	354	67
5% MgO 0.3:0.7 Asp:Glu	120	210	99	75
2% MgO 0.84 Asp	545	288	190	129
2% MgO 0.5:0.5 Asp:Glu	322	162	132	101

**Table 7:** Results of the antibacterial properties of the samples against Escherichia coli, adjusted for dilutions.

Sample	2 h	4 h	6 h	24 h
1% MgO 0.84 Glu	9.6 × 10^6^	7.8 × 10^6^	2.42 × 10^6^	1.22 × 10^6^
7.5% MgO 0.7:0.3 Asp:Glu	6.4 × 10^6^	4.32 × 10^6^	7.08 × 10^6^	1.34 × 10^6^
5% MgO 0.3:0.7 Asp:Glu	2.4 × 10^6^	4.2 × 10^6^	1.98 × 10^6^	1.5 × 10^6^
2% MgO 0.84 Asp	1.09 × 10^7^	5.76 × 10^6^	3.8 × 10^6^	2.58 × 10^6^
2% MgO 0.5:0.5 Asp:Glu	6.44 × 10^6^	3.24 × 10^6^	2.64 × 10^6^	2.02 × 10^6^

**Table 8:** Results of the antibacterial properties of the samples against Staphylococcus aureus.

Sample	2 h	4 h	6 h	24 h
1% MgO 0.84 Glu	147	141	75	8
7.5% MgO 0.7:0.3 Asp:Glu	160	90	98	57
5% MgO 0.3:0.7 Asp:Glu	92	120	84	35
2% MgO 0.84 Asp	95	89	130	80
2% MgO 0.5:0.5 Asp:Glu	130	75	86	51

**Table 9:** Results of the antibacterial properties of the samples against Staphylococcus aureus, adjusted for dilutions.

Sample	2 h	4 h	6 h	24 h
1% MgO 0.84 Glu	2.94 × 10^6^	2.82 × 10^6^	1.5 × 10^6^	1.6 × 10^5^
7.5% MgO 0.7:0.3 Asp:Glu	3.2 × 10^6^	1.8 × 10^6^	1.96 × 10^6^	1.14 × 10^6^
5% MgO 0.3:0.7 Asp:Glu	1.84 × 10^6^	2.4 × 10^6^	1.68 × 10^6^	7 × 10^5^
2% MgO 0.84 Asp	1.9 × 10^6^	1.78 × 10^6^	2.6 × 10^6^	1.6 × 10^6^
2% MgO 0.5:0.5 Asp:Glu	2.6 × 10^6^	1.5 × 10^6^	1.72 × 10^6^	1.02 × 10^6^

**Table 10:** Sample composition.

L.p.	*L*-Aspartic Acid, g: *L*-Glutamic Acid g	% Periclase Content
1	0.84:0	1
2	0.84:0	2
3	0.84:0	7.5
4	0:0.84	1
5	0.5:0.5	1
6	0.5:0.5	5
7	0.5:0.5	7.5
8	0.7:0.3	1
9	0.7:0.3	5
10	0.7:0.3	7.5
11	0.3:0.7	2
12	0.3:0.7	5
13	0.3:0.7	7.5
14	0.84:0	0
15	0:0.84	0
16	0.5:0.5	0
17	0.7:0.3	0
18	0.3:0.7	0

## Data Availability

Data available on request.
